# A culture of care: How Lotus House Women's Shelter heals program participants through genuineness, space, high expectations, dignity, individualized attention, and community

**DOI:** 10.1002/jcop.22579

**Published:** 2021-05-06

**Authors:** Asia A. Eaton, Dionne P. Stephens, Yanet Ruvalcaba, Jasmine Banks, Sundari Foundation Inc.

**Affiliations:** ^1^ Department of Psychology Florida International University Miami Florida USA; ^2^ Department of Psychology University of Michigan Ann Arbor Michigan; ^3^ Sundari Foundation, Inc. dba Lotus House Women's Shelter (Lotus House) Miami Florida USA

**Keywords:** ethics of care, gender, homeless persons, homelessness, organizational culture, women's shelters

## Abstract

The present study was designed to examine perceptions of Lotus House Women's Shelter from the perspective of former program participants, for the purpose of informing shelter programming and policies. Our qualitative research followed a community‐based participatory research framework. Fifty diverse women graduates of Lotus House Women's Shelter participated in eight focus groups to discuss their experiences with Lotus House and other shelters. Findings from this study highlight the elements that create a “culture of care” within an organization. Participants described Lotus House shelter culture as genuine, defined by dignity and respect, having high expectations for guest independence and accountability, giving space to rest and recuperate, recognizing and accommodating individual needs and experiences, and fostering a sense of community. Creating an organizational “culture of care” is an avenue by which any shelter or related organization can enhance the experience of program participants.

## 1 INTRODUCTION

Women and girls make up about one‐third of the homeless population in the United States (Moses & Janosko, 2019), and their experiences of homelessness differ from those of men and boys (Ferguson et al., 2015; Henry et al., 2019; Rew et al., 2008). For example, women experiencing homelessness more often have a history of abuse in childhood and adulthood (North & Smith, 1993; Ritchey et al., 1991), and are more likely to be victims of violence while homeless (Evans & Forsyth, 2004; Roll et al., 1999). Women also use unique strategies to survive, such as hiding their bodies and downplaying their visibility (Casey et al., 2008; Huey & Berndt, 2008), as well as using sex and intimate relationships for survival (Walls & Bell, 2011; Warf et al., 2013). In terms of consequences, women experiencing homelessness report experiencing greater safety issues than men (Jasinski et al., 2005; Milburn & D'Ercole, 1991) and, in some cases, more significant health consequences (Muñoz et al., 2005).

Gender differences in the antecedents, nature, and consequences of homelessness persist while individuals reside in the shelter system (de Vet et al., 2019) and in permanent supportive housing (Winetrobe et al., 2017). In randomized controlled research on 18 shelters across the Netherlands, de Vet et al. (2019) found women had lower social and societal embeddedness than men before and after their transition from shelters to community living, including lower satisfaction with living conditions, and lower levels of self‐regulation than men. These women were also less likely to be educated, more likely to be unemployed, more likely to have been victimized, less satisfied with their health and empowerment, lower in self‐esteem, and higher in psychological distress than their male counterparts (de Vet et al., 2019). This study concluded that women in homeless shelters are especially vulnerable and need shelter experiences that support their biopsychosocial well‐being. Therefore, understanding the benefits and downfalls of women's shelter experiences is critical for addressing their needs and supporting their transition out of homelessness.

### 1.1 Women's shelter experiences

Past research on women's experiences with homeless shelters uncovers both drawbacks and benefits. In a scoping review of research on women experiencing homelessness, women reported feeling “let down” by the attitudes and behaviors of homeless service providers (Phipps et al., 2018). For example, women described a lack of respect from U.S. service providers, including feelings of being stereotyped as “bad mothers” by family shelter staff in California (Reppond & Bullock, 2020) or as drug addicts at a homeless family shelter in the Midwest (Sznajder‐Murray & Slesnick, 2011). Another set of studies in Ireland and the United States detailed how women in a variety of homeless service settings felt talked down to and treated like children (Mayock et al., 2015; Reppond & Bullock, 2020). Other work on women experiencing homelessness in Chicago and Detroit found that shelter staff failed to inquire about women's history of violence victimization (Huey et al., 2014), missing the opportunity to recognize women as individuals with unique needs and histories. These women also said they were not asked to describe the mental and physical health consequences of their victimization (Huey et al., 2014).

Pertaining to shelter operations, women experiencing homelessness have described limited access to services and a lack of coordination among the numerous services offered (Hamilton et al., 2012). Guests also described difficulty obtaining information about available programs and resources (Hamilton et al., 2012; Huey et al., 2014). Other negative attributes of the shelter system were the bureaucracy and strict system of rules seen at one faith‐based homeless shelter located in the Midwest (DeWard & Moe, 2010). At another religiously based homeless shelter in the Midwest for single women and families, older women described the shelter as confusing and chaotic (Davis‐Berman, 2011), saying they had to “go through 10 steps just to get one thing” (p. 366). At a winter emergency shelter in the United States (Biederman & Nichols, 2014), women described receiving conflicting information about when and how to gain entry. Research also found that older women at shelter locations across Canada wanted the shelters to be more comfortable and less institutionalized (McLeod & Walsh, 2014).

Some studies, however, hold more hope for the potential of shelters to support the healing and recovery of women experiencing homelessness. In a study of mothers in two “strength‐based” shelters in Northeastern United States, participants expressed that the shelter staff was supportive (Cosgrove & Flynn, 2005). In these more successful shelters, the staff used feminist models of intervention that emphasized women's strengths and competencies. In another study, service providers to women victims of violence were rated more highly by shelter participants than homeless adult and youth service providers, perhaps because of the targeted nature of the services and community (Asmoredjo et al., 2017). Finally, some women have had the experience of feeling competent and autonomous in shelters. In a study of sheltered mothers in the Czech Republic, women were able to reframe their experiences from being passive victims to survivors (Glumbíková & Gojová, 2020).

### 1.2 Shelter culture

Much is known about the pros and cons of specific shelter services, such as child care (Dashora et al., 2012), mental health counseling (Sleath et al., 2006), employment services (Radey & Wilkins, 2011), health care (Wenzel et al., 2001), drug and alcohol treatment (Slesnick & Erdem, 2013), and case management (Heslin et al., 2003a). But the context in which these services exist, or the culture of the shelter, may be equally important for women's health and well‐being. Indeed, many of the negative and positive shelter experiences described in the previous section were directly related to organizational culture, such as having supportive staff who spend time finding out about participants' lives.

Organizational culture includes underlying beliefs and assumptions, shared values, and ways of interacting that contribute to the organization's social and psychological environment (Schein, 1992). Organizational culture is enduring, being rooted in fundamental values and beliefs (Chatman & O'Reilly, 2016). These may include things like the importance of customer focus, teamwork and cooperation, or adaptability (Denison & Mishra, 1995). A strong sense of shared values, attitudes, and practices indicates the organization is a bounded unit, and provides it with a distinct identity that influences the behavior of all members (Schein, 1985).

Several validated measures of organizational culture exist, which have been used for a variety of purposes, such as to evaluate and create organizational change (e.g., Gaucher & Kratochwill, 1993). However, existing measures of organizational culture have typically been developed, tested, and validated on for‐profit businesses rather than service organizations such as homeless shelters. The highly popular organizational culture profile measure, for example, has been used to understand the culture of government agencies (O'Reilly et al., 1991), public accounting firms (Sheridan, 1992), private consulting firms (Adkins & Caldwell, 2004), and technology firms (Hartnell et al., 2016). These businesses differ from nonprofit organizations in countless ways, such as their size, mission, funding source, and social status.

While some nonprofit institutions have been examined using validated organizational culture measures, such as high schools and universities (Erdogan et al., 2006; Erkutlu, 2011), these also vary from shelters in the populations they serve and the goals of the organization. Thus, the culture of a successful shelter may not look like the cultures that have been established in other for‐profit or non‐profit organizations—even if these varied organizations have cultural elements that could be usefully transferred among one another. Therefore, in our investigation of shelter organizational culture, we took a bottom‐up, qualitative approach to understand the culture of one specific successful women's shelter in Miami, Florida, namely the Lotus House Women's Shelter (Lotus House), which boasts a success rate of over 80% for program participants.

Organizational culture is a concrete and actionable aspect of shelters because not all shelter services and affordances may be transferrable to other shelter environments. All shelters, however, have an organizational culture that can be evaluated and improved (Alvesson & Sveningsson, 2016). Aspects of shelter culture that have been described favorably by program participants include a culture of humane treatment, evidenced by staff that are friendly, nonjudgmental, and considerate (Kerman et al., 2019). On the other hand, some shelters lean more toward a culture of social control in policies and practices, being focused on changing or controlling the behavior of individual women (Hartnett & Postmus, 2010). For example, in a study of 97 Ohio agencies providing services to women experiencing homelessness or domestic violence (Hartnett & Postmus, 2010), researchers found many shelters controlled women's daily activities, with 84% of shelters requiring women to participate in case management and 55% requiring them to undergo drug and alcohol counseling. Another example of social control can be seen in how shelters use eligibility criteria to categorize women and direct services (e.g., as abused teens, drug addicts, survivors of violence, etc.; Williams, 2016).

Studying the culture of shelters is a critical next step toward understanding and addressing homelessness, as shelter policy and program factors are seen as playing a strong role in participant outcomes (McDivitt & Blasco, 2015). For example, shelters that use trauma‐informed models have better outcomes than “treatment as usual” for participants (Hopper et al., 2010). Indeed, organizational culture is generally known to influence the beliefs, attitudes, and behaviors of those in the organization, with strong and positive cultures leading to improved outcomes for organizational members and organizational performance (e.g., Hartnell et al., 2019). In sum, the main research question for the present study was “How do graduates of Lotus House shelter perceive the shelter culture?” The inductively derived answer to this question was expected to shed light on the reasons for the shelter's success, which could be transferred to other shelters and contexts.

## 2 METHODS

### 2.1 Lotus House

All study participants were women graduates of Lotus House. The largest women's shelter in the State of Florida, Lotus House provides emergency shelter with comprehensive supportive services to assist women, youth, and children experiencing homelessness. Lotus House offers holistic wraparound support services using a gender‐specific, trauma‐informed framework. The services provided include access to health and mental health services, evidence‐based group and individual counseling, evidence‐based individual assessments for children and child and family therapies, parenting courses, resource coordination, employment and educational supports, life skills and job readiness training, alternative pathways to healing, and more. Lotus House also has a reputation in the local community, and among those experiencing homelessness in South Florida, as having a supportive culture (Smiley, 2016).

Founded in 2006, Lotus House is located in the heart of the historic African American district of Overtown, Miami—one of the poorest neighborhoods of Miami, itself one of the poorest large cities in the United States. In that time, Lotus House has grown from one small building sheltering 34 women to its current capacity of over 500 women, youth, and children nightly. At full capacity throughout the year despite the pandemic, Lotus House sheltered 1354 women, youth, and children in 2020. Today, Lotus House occupies a state of the art, comprehensive homeless services facility, called Lotus Village, which includes four floors of residential programs with supportive services nodes for programming, counseling, and resource coordination on each, an intake sanctuary, children's wellness center, professional culinary center and training program, education and employment center, salon, yoga and exercise room, art and activities lab, children's play rooms, urban gardens, and a neighborhood health clinic.

Demographically, those sheltered by Lotus House have historically been 65%–70% African American/Black, 25%–35% Hispanic, and 1%–4% Other. Most of the women and children who enter Lotus House are referred by the City of Miami Homeless Outreach teams, a program of the Miami‐Dade County Homeless Trust. Lotus House also serves as a direct access point for youth ages 18–25 and domestic violence victims. Approximately 99% of the women served at Lotus House have histories of domestic and/or intimate partner violence, are victims of other gender‐based violence and crimes, and/or have other significant trauma.

Lotus House data from 2018 to 2020 reveals the pervasive traumatic life events experienced by sheltered women, including domestic/intimate partner or family violence (78%), physical assault (31%), sexual assault as an adult (10%), human sex trafficking (4%), violent crimes, or other severe traumas. The sheltered women at Lotus House are not only challenged with histories of gender‐based violence and trauma, but many of them cope with disabilities. Between 2018 and 2020, approximately 85% (1114 out of 1311 women served) of the program participants^1^ suffered from a disability.

Recognizing the fragility of the women and children who enter its doors, Lotus House identifies the special needs of each program participant by conducting a preliminary clinical assessment at intake. The women, then, mutually create a personalized action plan consisting of goals, action steps, and resources to help them make the transition to safe housing. To ensure the safety and well‐being of others, program participants are also asked to agree to follow the policies implemented at Lotus House, which include among other things adherence to non‐violence principles; abstinence from the use of non‐prescription drugs or alcohol of any kind; maintaining their living space in a clean, neat, and orderly manner; and the “Savings Rule^2^.” To maintain a drug‐free environment, program participants agree to be tested at entry and randomly thereafter and those with substance abuse histories participate in individual counseling and recovery support groups. An intervention by a trained team member occurs if the tests results are positive.

The present study was conducted to examine the experiences of a diverse group of women program participants in Lotus House, for the purpose of informing future programming and policies at Lotus House and related organizations. In our analyses, we focused on perceptions of the organizational culture at Lotus House among Lotus House “alumni” (the term used at Lotus House for former participants) who successfully transitioned from the shelter to stable housing. Over 80% of those sheltered by Lotus House successfully exit to homes outside the shelter system, including permanent supportive housing, independent living, relocation or reunification with friends and family, assisted living facilities and, as needed, long‐term residential care.

### 2.2 Participants

Fifty women graduates of the Lotus House participated in eight focus groups; these focus groups served as the unit of analysis, with all reported organizational culture themes being present across multiple groups. Of these, 40 completed paper‐and‐pencil demographic surveys. Participant ages ranged from 27 to 66 (*M* = 53.28, *SD* = 10.03). Seventy percent (*n* = 28) identified as Black or African American, 17.5% as White (*n* = 7), 10% as Hispanic/Latina (*n* = 4), and 2.5% as Black/Hispanic (*n* = 1). Of those who responded to the question about their sexual orientation (*n* = 27), 88.89% self‐identified as heterosexual (*n* = 24), and 11.11% self‐identified as Lesbian/Openly Gay (*n* = 3). Of those who provided a numerical response for “Current Income” (*n* = 18), the average amount of monthly current income was $595.32 (*SD* = $649.56). Participants' average length of stay at Lotus House was 11.21 months (*SD* = 5.97). All participants in this study stayed at Lotus House without children. All participants successfully graduated from the program, representing the majority (over 80%) of all program participants at Lotus House. Unfortunately, it was not possible to recruit unsuccessful graduates as we did not have their contact information. As with all extremely impoverished populations, many are transient, unable to afford phones, and it can be difficult to impossible to track their long‐term whereabouts after exit.

Participants were recruited through the Lotus House Project Coordinators, who placed individual calls to known alumni inviting them to participate. Each focus group took place at Lotus House. Participants were given $25 Target gift cards as incentives, as well as provided breakfast or lunch, depending on the time the groups took place.

### 2.3 Researchers

The main research team included a White female Associate Professor (the first author), a Black female Associate Professor, a Latina Ph.D. student (the third author), a Black and Taiwanese female undergraduate student (the fourth author), and Black and White female members of the Lotus House staff (the fifth author). Among this racially diverse group of women were individuals who had experienced homelessness, individuals in recovery from drug and alcohol addiction, Lesbian, Gay, Bisexual, Transgendered, and Questioning—LGBTQ + individuals, and individuals with personal and family histories of abuse, domestic violence, trauma, and mental illness. We recognize, however, that despite having some shared experiences with participants in this study, some of us held tremendous educational and financial privilege relative to the average study participant.

### 2.4 Procedure

Our research followed a community‐based participatory research model (CBPR; Vaughn et al., 2018) in which Lotus House community members, including leadership and former shelter program participants, worked collaboratively and iteratively with the researchers to shape the research questions, methods, measures, and findings, including co‐authoring the current paper. Guided by the assumption that social change is more likely to occur when nonacademic researchers equally participate in the research process, CBPR centers mutual learning, participatory practices, co‐construction of knowledge and action that benefits the target study population and their communities (Baum et al., 2006; Israel et al., 2001; Rosenthal et al., 2014; Vaughn et al., 2018). Further, scientific rigor improves when nonacademic researchers have decision‐making and agency to engage across the research process (Baum et al., 2006; Newell & South, 2009; Vaughn et al., 2018), congruent with the scientific principle of critical multiplism (Shadish, 1993).

The first step in our partnership was determining what questions Lotus House wanted help answering. Among other things, Lotus House wanted to ascertain perceptions of the most and least helpful elements of program participants' experiences, how Lotus House compared to other shelters, and what Lotus House could improve upon for future program participants. In‐person discussions of the topics and questions of importance to Lotus House took place regularly over the course of a year at the Lotus House campus. During this introduction time the research team learned about Lotus House's operations, goals, and values, and developed a trusting relationship with Lotus House staff and leadership.

Next, to achieve an equal partnership in research development, processes, and products, we co‐created a four‐page Memorandum of Understanding (MOU) that outlined Lotus House's equal status in all research endeavors, and the research team's commitment to honoring the values and principles of Lotus House. In terms of honoring the values of Lotus House, the MOU outlined, for example, that “PWR Lab and Lotus House agree to carry out the research in a trauma‐informed, kind, compassionate, respectful and collaborative manner in accordance with a mutually agreeable timeline of activities.” In terms of power and resource sharing, the MOU outlined that “PWR Lab agrees to share with Lotus House all information, data, abstracts, summaries, analyses, draft manuscripts, draft papers, and all other extracts…; in no event shall any of the foregoing be publicly disseminated or published, in any form or by any means of communication, by PWR Lab, without the prior written consent and agreement of Lotus House (which may be a conditional consent or agreement), in its sole discretion.”

After the creation and signing of the MOU, we began co‐creating discussion guides for the present study. These were developed using practical questions of importance to Lotus House staff and alumni regarding the efficacy of various components of their shelter over time. While we did not originally set out to map the organizational culture of Lotus House, the dimensions of Lotus House's organizational culture fell naturally from the questions about the pros and cons of Lotus House. These questions were complemented with theoretically driven questions from existing research on women's experiences with shelters in the United States.

The question set was then modified through a series of in‐person meetings with the Lotus House Executive Director, Health and Wellness Director, and Project Coordinator. After this, the focus group questions were pre‐tested on a sample of Lotus House staff who were former program participants of Lotus House, and additional adjustments to language and content were made. Additionally, two organizational staff members served as cultural consultants; specifically, they drew upon their decades of experience working with women experiencing homelessness and within shelter settings to ensure that the procedures and research tools were culturally appropriate. They also assisted in guiding the researchers' own reflexivity processes regarding their roles, power, and influence as outsiders to this community and experience (Corbett et al., 2007). This was achieved through regular team meetings throughout the study design, implementation, and data analysis phases. The full set of focus group questions is available in the study Appendix.

This study included eight focus groups, which lasted on average 1 h and 25 min. Focus groups were conducted in a private meeting room within the shelter. Audio recordings were transcribed by three trained undergraduate female research assistants. All groups were facilitated by a single moderator, either the first or second author. After obtaining participants' consent, the discussions were recorded on digital voice recorders. The groups were conducted in English, although participants occasionally used Spanish or Creole phrases or terms. Ethical clearance was granted by the Institutional Review Board of the Principal Investigator (PI)'s university.

The focus group discussions explored women's experiences and attitudes with shelters in South Florida, including but not limited to their time as program participants at Lotus House, and their experiences of street homelessness. In a total of 12 questions, participants were asked about their experience at Lotus House, their time spent at other shelters (if any), the challenges they faced while homeless, their coping and safety strategies while homeless, what the ideal shelter would look like, and how Lotus House could improve. Given the sensitivity of the topic, several steps were taken to protect participants' privacy and create an atmosphere of comfort and trust. The use of pseudonyms on large name tags ensured that personal name identification was avoided. Another step to ensuring privacy was holding the focus group in a commonly used meeting room to avoid identification of the study purpose to non‐participants. Drawing upon CBPR examples, this including having trusted Lotus House staff members lead the recruitment and introductions between staff and research team members to increase the credibility and importance of the work for benefiting women experiencing homelessness (Corbett et al., 2007).

The use of focus groups allowed for gaining insight into various perspectives from the members about their attitudes, beliefs, and experiences at Lotus House. The focus groups allowed for authentic experience and dialogues to be captured. Focus groups also capture information from a group dynamic that other methods do not capture (Gibbs, 1997). For example, participants were able to reflect, recall, and expand on common experiences that were brought up by other participants such as experiences with specific shelter staff members, and opinions about shelter services, and so forth. Sessions began with a buffet lunch with participants and team members in a circular seating set up; this allowed for informal conversation and equitable interactions in the space (Corbett et al., 2007; Ross, 2017). Finally, research team members also shared their level of knowledge about the study topic, including their gaps in knowledge, to ensure participants were centered as the experts of the topic of interest (Baum et al., 2006).

### 2.5 Analyses

Thematic analysis was conducted on the interview transcripts using guidelines established by Braun and Clarke (2006). Thematic analysis is a well‐established qualitative method that enables a flexible analysis of complex data by reducing the data into themes (Braun & Clarke, 2006; Crowe et al., 2015). The purpose of thematic analysis is to delineate participants' subjective feelings, thoughts, or behaviors with accuracy. Consistent with thematic analysis, we identified themes related to organizational culture that strongly emerged from the data, and did not try to fit them into a pre‐existing framework for organizational culture (Braun & Clarke, 2006; Patton, 1990).

The data were coded by two coders, the third and fourth authors, trained by the first author, using Microsoft Word 2017. First, two focus group transcripts were selected at random and read individually by the two coders and the first author. These authors separately identified initial codes related to organizational culture that emerged from the transcripts. These initial codes were discussed together, and an initial codebook was created. Each of the two coders then separately read and coded two additional focus group transcripts using the initial codebook. In a second meeting, this coding was compared and contrasted, and the codebook was updated based on insights from discussion. The final, updated codebook was then used by the two coders to code two more transcripts. The coding from these transcripts resulted in an interrater reliability of *κ* = 0.83. All disagreements were resolved through discussion. Given that the high interrater reliability at this stage (Belotto, 2018), the two coders moved to code the remaining two transcripts, and then returned to code the first four transcripts using the final codebook. Inconsistencies between the themes were addressed together until there was 100% agreement, resulting in six organizational culture themes.

## 3 RESULTS

Analyses revealed a “culture of care” from within the organization. The “culture of care” was comprised of six themes, each of which highlighted aspects of interpersonal treatment: (1) *Genuineness*, (2) *Space to rest and recover*, (3) *Expectations for independence and accountability*, (4) *Being treated with dignity and respect*, (5) *Individualized attention and care*, and (6) *Community orientation* (Table 1).

**Table 1:** “Culture of care” themes

Coding Theme	Description
(1) Genuineness	Shelter leadership and staff having authentic and transparent interpersonal motives that were non‐transactional
(2) Space to rest and recover	The institution providing sufficient space and time for program participants to heal
(3) Expectations for independence and accountability	Leadership and staff holding program participants to high but achievable standards, including following basic rules and developing independent living skills
(4) Being treated with dignity and respect	Leadership and staff treating program participants with respect and humanity
(5) Individualized attention and care	Staff providing one‐on‐one support to program participants
(6) Community orientation	Program participants feeling included and part of a community

### 3.1 Genuineness

*Genuineness* was defined as shelter leadership and staff having authentic and transparent interpersonal motives that were non‐transactional. Participants specifically mentioned that Lotus House was different from other shelters because Lotus House staff did not want anything from the participants.

You know I was given a startup bag which is a bed in a bag, personal hygiene, clean towel—pretty colors, pretty washcloth and I'm like “they want something”. But at the end of the day didn't want nothing they didn't want nothing except for me to rest and get my life in order and they actually was truthful about it. (“Lola”)

Participants also reflected on how Lotus House genuinely cared about their well‐being and recovery:

So basically the difference between other shelters and Lotus House is that they give you a little bit more time and they value you. When I first came to Lotus House the biggest difference was that I felt that they cared… (“Kim”)

When I got to the Lotus House and they handed me the covers and they're like, oh you, this is yours, this is yours, this is yours and you don't have to worry about it. (“Trish”)

At other shelters, involvement in religious events, following strict rules limiting independence, or quick full‐time employment, are required of participants to access services (Dickinson et al., 2017). For example, faith‐based programs, which constitute a substantial portion of the services system for people experiencing homelessness (Heslin et al., 2003b), can be controlling and demanding, requiring members to profess a particular faith and/or attend religious events to qualify for services (Mulder, 2004). Participants mentioned this explicitly when comparing Lotus House to other shelters:

It was too much rules being thrown at you [in the other shelter]. Way too much rules. Lotus House got rules too, but not like that. (“Ekkie”)

### 3.2 Space to rest and recover

The *space to rest and recover* theme was defined as the institution providing sufficient space and time for program participants to heal. In the following quote, “Kim” reflects on how the organizational culture of Lotus House gave her the freedom to take care of herself and psychologically and physically relax.

Because that's what you need after it's like you take a sigh of relief it's like I was holding my breath for so long and I could relax and just de‐stress for a few days; that is very important you know, those two things, being valued and actually feeling like people care and being able to actually relax like you are in a good safe space. (“Kim”)

“Milagros” echoed this sentiment, noting that she was able to clear her mind at Lotus House thanks to the space and environment they provided:

Lotus House they gave me an opportunity just to—to have a clear head and like you know sit in the garden and just meditate. And just think about whatever you need to do. Not stressing on what I had to do. But, just preparing me for what I need to do and having life outside the shelter. (“Milagros”)

“Dominique,” meanwhile, pointed to Lotus House's long‐term shelter stay as providing sufficient time to rest and recover from her experience of homelessness.

…you know like both of the ladies said, truth is, unlike most shelters, you're there roughly 2 months they may give you 3, depending on your case. But that's it. You're talking about an unrealistic idea that, okay, within these 2 months, or 3 give or take, you're gonna find a job, find a place, save some money along the way, and go. And, it wasn't ideal so when I got to Lotus House back then it was a year. Or roughly up to a year. (“Dominique”)

### 3.3 Expectations for independence and accountability

*Expectations for independence and accountability* was defined as leadership and staff holding program participants to high but achievable standards, including following basic rules and developing independent living skills. “Nikki” explained that being expected to follow rules prepared her for living in her own apartment:

And one of the things that Lotus House taught me, which I'm so grateful for, is how more, how to follow rules. I mean really truly you gotta follow the rules and new direction. I was just beginning to learn to follow rules. Lotus House, they instill it into me. So then I was ready to go to my own apartment. (“Nikki”)

Others discussed how the expectations for program participants at Lotus House were reasonable and fair, especially in comparison to other local shelters.

…just as Delta said, long as you follow the rules, long as you comply that's…you know what I'm saying, you was fine. That's what I did back then. I didn't care for the 6 o'clock curfew, even though I care to be out like that, but I obliged…you know I obliged by. When I worked, cause I was on call working at Macy's, so when I had to go, the only exception was if you had to work that was it. Otherwise, they were…they was strict, sorta halfway when it came to the curfew, but they weren't as bad. (“Dominique”)

Where? (“Lisa”)

Here! (“Rosie”)

Here? (“Lisa”)

Yeah, here. Cause, [name of other shelter] you know, if  theirs was 7 o'clock, back then, and you better have come around hoping and praying as to why you need to come back in, cause most of the time you couldn't. At the same tone I mean, like I said, unlike here, it was like depending on circumstances but usually, they were pretty decent with the curfew. (“Dominique”)

Lola explained that the culture of Lotus House supported her in being more accountable, a trait that she internalized and now takes pride in.

You made a commitment (“Rai”)

You made a commitment you need to honor it. That's what it gave me too. I am like a woman of my word. If I said I'm going to be there, I'm going to be there. If I say I'm going to do it, I'm going to do it. (“Lola”)

Another participant explained that the services Lotus House provided supported her independence:

I mean, they tried to establish you so when you leave Lotus House you can stay self‐sufficient…. I'm totally self‐sufficient now and I live in Independent Structures Living…They taught us how to budget our money, I took a food class and graduated, I got a certificate, we had what was the thing? Body talk is that what it was called? Body Talk? Yeah, we had Body Talk, we had aerobics, we had arts and crafts. (“Lisa”)

### 3.4 Being treated with dignity and respect

*Being treated with dignity and respect* was defined as leadership and staff treating program participants with respect and humanity. While participants did not explicitly use the terms “respect” and “dignity” in recounting their experiences with the shelter environment, several specific examples made these clear. First, “Betty” described being listened to deeply by the staff, and not interrupted in telling her personal story upon intake:

When I did get the interview with her, and she said tell me about yourself and I hate talking. God. And I went to talking and so she—she just look and she never interrupt me she would let me run my mouth, and she said: “do you need to tell me anything else?” (“Betty”)

Another example came from “Renee,” who recounted a time that staff members apologized to her after they made a mistake:

My drug test came up positive for methamphetamine. It was my fault. Because she gave me Zantac, it was a false positive. The psychologist figured it out. So the psychologist and director apologized to me. (“Renee”)

“Kim” described being treated with humanity.

I wasn't just a number. It was a different experience. (“Kim”)

The respect Lotus House showed to participants was also mutual, with participants expressing a great deal of respect for staff leadership:

…it brings tears to my eyes cause like [leader name] is really, really awesome… I remember one time I asked her, I said, what made you decide to open a shelter? Because she was a lawyer. Like she, she's like corporate lawyers and everything, and I like, I had the utmost respect for her ever since she said because it was, I was 13 years old and I went on a trip to New York with my friend and her family, and we were walking along the street and I saw a woman, you know, digging in the trash and people who are just passing by her, but she didn't matter. She was cold and she, you know, she was just really, really just, no, she just looked out of it. I just, it stuck with me all of these years…(“Yubraska”)

### 3.5 Individualized attention and care

The *individualized attention and care* theme was defined as staff providing one‐on‐one support to program participants. Beyond treating participants with dignity/respect and authenticity, staff took the time and effort to engage with and support the specific needs of each shelter participant. With “Sweet Peaches,” this took the form of shelter leadership writing personalized letters on her behalf to the court system:

Seriously, I really respect her for stuff she has done for me. You know, while I'm away, helping me. You know, writing letters to the judge, trying to help my attitude in here… (“Sweet Peaches”)

Another participant explained that Lotus House staff made appointments for her in her job search:

…and by the time and I never forget looking for a job and Chef would be helping me looking for a job and Miss Rai would be getting me into the appointment and stuff like that… (“Lola”)

“Lisa” explained that she could still rely on her individual relationships with Lotus House staff even now that she is an alumni:

I feel like I can always come to Lotus House If I ever need it. Like if I need to talk to somebody, I mean, you know, mentoring or counseling or something, I can go to [leaders and staff members] or anybody and say, listen, I need to talk to somebody. You know, before, I don't want to make a bad decision. I'm scared. You know what I'm saying. (“Lisa”)

Finally, “Caveman Mama” explained that she was personally looked after by the Lotus House founder and CEO:

You know, when I came here, [leader name] made sure that I went to rape treatment counseling at the hospital, she made sure that I ate, she made sure that I wasn't eating junk. She just made sure I was going to my appointments, stuff like that, you know. (“Caveman Mama”)

### 3.6 Community orientation

*Community orientation* was defined as program participants feeling included and part of a community. Participants described Lotus House as being a place where they felt deep and life‐long belonging.

Thank you! Okay. This is what, this is what Lotus House is. A place where you can go to and where you can redeem yourself. A place where you can go when you need help. A place that's not just a shelter, it's home. Inside it has walls and its beautiful women and children. There is not place on earth that I would rather be, with no offense to Mister Walt Disney, although I really love Mickey Mouse. Lotus House. The best place on earth is Lotus House. (“Lisa”)

…we used to, everybody used to look out for each other. We had the maternity wing, the single wing and…we all checked on each other, cared about each other. (Ekkie)

Participants also mentioned that the all‐women nature of Lotus House allowed for close female bonding:

…once I came here it was like I said, it was sisterhood for me. It was the sisterhood. (“Deborah”)

But they made me feel welcome and the hospitality was off the charts! By the time I was going and meeting people, I said I'm in the shelter down the street, you know Lotus House? They dropping me off and coming to see me. They—I don't know how that dissipated that embarrassment, that feeling. But I was glad to be part of Lotus House. Lotus House girls! (“Sharonne”)

(Following this, the entire focus group spontaneously chanted in unison “Lotus House girls!”)

Not all participants, however, felt naturally strong connections to the community. For some participants, having roommates and being in a highly social, all‐female environment were challenges that Lotus House helped them navigate:

Just to calm myself. That's what I learned in therapy at Lotus house. You know, you laugh, talk to yourself, you know you walk away and that's what this taught me. But like I said, most taught me how to have a lot of patience and listen… There is a reason behind why they're acting a fool. (“Yubraska”)

It teaches you a lot of patience with women in here. Yeah. (“Ekkie”)

Because I'm the type person, I do not like crowds. I don't want to like. (“Yubraska”)

Like I don't like being around other females. (“Ekkie”)

Like, I want to stay out of drama. If I could stay out of it, I would stay out of it. But I don't want to be in drama and yes, when you're around a whole bunch of females. (“Yubraska”)

## 4 DISCUSSION

Findings from this study highlight the essential elements that create a “culture of care” within an organization. In the case of the Lotus House Women's Shelter, program participants reported that this culture enabled them to heal from trauma and return to lives of independence and civic participation. Former participants of the shelter described the shelter culture as genuine, defined by dignity and respect as well as high expectations for independence and accountability, somewhere to rest and recuperate, a place where individual needs and experiences were understood and met, and a place of belonging and sisterhood.

Our concept of an organizational “culture of care” has overlap with the concept of an “ethics of care” from social work, nursing, and philosophy (Edwards, 2009); indeed, work on an “ethics of care” (also known as “care ethics”) inspired our nomenclature. The concept of an “ethics of care” grew out of feminist philosophy as an “alternative moral epistemology” (Walker, 1989, p. 15) in which moral action and thought was centered around relationships rather than rights. Demonstrating this, participants did not describe Lotus House in terms of the rights it afforded them, the justice it served, or principles maintained; instead, participants described their interactions with Lotus House in terms of nurturance, community, mutual respect, and genuine connection. Moreover, the rights and obligations afforded Lotus House program participants were accepted and internalized because they came from a place of caring. Consistent with care ethics, members of a culture of care are attuned to others in their professional environment, and care for others as both a social and political practice as well as a virtue and habit (Leget et al., 2019). While some work has already described attempts at practicing an “ethics of care” in homeless shelters (Ranasinghe, 2017), this study was done in a men's shelter, and may not reflect the nature of the care needed or provided in an all‐women shelter.

### 4.1 Review of “culture of care” themes

#### 4.1.1 Genuineness

The first element of organizational culture revealed in the data was that of genuineness. The theme of genuineness was defined as Lotus House Shelter leadership and staff interacting with program participants authentically and without strings attached. The culture of Lotus House was not “exchange oriented,” in which benefits were given with the expectation of returns (Clark & Mills, 1993), but based on genuine care and a felt responsibility for meeting the needs of participants. Research on organizational culture finds that authentic leadership and culture are assets to organizations, having positive effects on follower attitudes (Azanza et al., 2013) and well‐being (Ilies et al., 2005). Authentic and non‐transactional social interactions may be especially important for guest well‐being in shelters, as people who experienced homelessness may be accustomed to usury and exchange‐based relationships with peers (Flåto & Johannessen, 2010) and performative help from organizations (Lancione, 2013).

#### 4.1.2 Space to rest and recover

Graduates of Lotus House also reported benefitting from temporal, psychological, and physical space to recalibrate and recover after their experiences with homelessness. First, participants discussed the importance of long‐term stays at Lotus House. Indeed, the average stay at Lotus House among the participants in this study was 11.21 months, far exceeding the average of 2 months at shelters nationwide (McDivitt & Blasco, 2015). Second, participants described psychological space, made possible in part by physical spaces like the meditation garden, that enabled them to clear their minds and pause from the fear and worry that characterizes life for women experiencing homelessness (Coston & Finckenauer, 1993).

#### 4.1.3 Expectations for independence and accountability

The next element of organizational culture articulated by Lotus House program participants was the high but achievable behavioral standards at Lotus House. This included asking participants to follow reasonable rules and directly and indirectly supporting them in becoming independent. While the rules at other shelters were described as overwhelming and unfair, Lotus House's rules were reasonable, but serious. Participants further explained that abiding by these rules developed their self‐esteem and enabled them to become self‐reliant. Research on homelessness finds increases in self‐efficacy are related to readiness for independent living (Epel et al., 1999) and active coping (Nyamathi, et al., 2000), making self‐reliance an important step in these women's transition from homelessness.

#### 4.1.4 Being treated with dignity and respect

Being treated with dignity and respect was exemplified by deep listening, acknowledgment of when the participant was in the right, and humanization. This respect from staff and leadership is contrary to other accounts of shelter systems, which describe strong power differences between participants and staff and demeaning treatment (DeWard & Moe, 2010). Indeed, research finds that people experiencing homelessness are among the most frequently dehumanized groups, being denied humanity and treated like objects or numbers (Harris & Fiske, 2006).

#### 4.1.5 Individualized attention and care

Being recognized and cared for as individuals has been found to be a source of dignity for guests and alumni recruited from a not‐for‐profit organization serving meals to men and women experiencing homelessness in Chicago, Illinois (Miller & Keys, 2001). In addition, individualized consideration is a key component of a highly effective form of leadership—transformational leadership (Northouse, 2016). Individualized consideration means demonstrating high concern for followers, treating them as individuals, getting to know them, and listening to their concerns and ideas (Judge & Piccolo, 2004). Research over the years has shown that transformational leadership is related to a number of positive organizational outcomes, including motivation, performance, and satisfaction of the organizational members, especially toward their leader (Bass & Avolio, 1994). In this sample, the Lotus House program participants consistently expressed their admiration for staff leadership, whose leadership style strongly influenced the shelter culture.

#### 4.1.6 Community orientation

Finally, program participants described feeling a “psychological sense of community” at Lotus House (Nowell & Boyd, 2010). A psychological sense of community is defined as including a feeling of belonging or relatedness, a sense of mattering to the group, a feeling that group members' needs will be met, and a shared emotional connection (McMillan & Chavis, 1986). Psychological sense of community correlates with many positive outcomes for organizational and residential members, including community participation (Talò et al., 2014), intention to stay in the residence (Perkins et al., 1990), and increased health and well‐being (Coulombe & Krzesni, 2019). Program participants' feeling that they were a part of a prideful sisterhood, one that they could call home and always return to, communicates all the elements of a psychological sense of community.

During the course of the interviews, participants also provided suggestions for ways they believe that Lotus House can improve. For example, “Deborah” suggested having successful graduates of Lotus House come back to visit for an “alumni day” and to meet and speak with current guests. “Moon Child” suggested more mentorship for younger women and women who have young children. “Betty” suggested providing more structure and programming for older women program participants such as specific counseling, as they have experiences that are different than those of younger women in the shelter. Multiple women suggested that the shelter give each woman a “chore” to teach responsibility while contributing to the cleanliness of the shelter, to provide earlier curfews, and to have a stricter dress code for Lotus House guests.

### 4.2 Practical implications

Creating an organizational “culture of care” is an avenue by which any shelter or related organization, irrespective of size, infrastructure, and financial resources, can enhance the experience of program participants through positive and genuine interpersonal treatment from leadership and staff. Our definition of a “culture of care” has implications for staff and leadership hiring and training, as well as for the creation of shelter policies, programs, and philosophies. First, shelter leaders and staff should be selected and trained on transformational leadership principles, including idealized influence, inspirational motivation, individualized consideration, and intellectual stimulation (Bass & Avolio, 1994; Northouse, 2016). These leadership dimensions are especially aligned with the culture of care elements of *genuineness*, *sense of belonging*, *individualized attention and care*, *expectations for independence and accountability*, and *being treated with dignity and respect*.

Second, shelter policies, programs, and principles should adopt a person‐centered approach in which program participants are treated with unconditional positive regard, empathetic understanding, and congruence (Hazler, 2016)—the core conditions of person‐centered therapy. Specifically, staff and programming should take an accepting and non‐judgmental approach to program participants, should communicate their desire to understand and appreciate the participant's perspective, and should be willing to relate to participants transparently without a façade. These conditions highlight the inherent dignity, autonomy, and value of participants, consistent with the elements of the culture of care.

Lotus House participants described being deeply grateful for their long‐term stay at Lotus House. Unfortunately, the number one reason for short shelter stays, and for why individuals experiencing homelessness do not seek shelters to begin with (McDivitt & Blasco, 2015), is lack of available beds. In 2019, the total population of people experiencing homelessness in the United States exceeded the number of available beds by over 257,000 (Statista, 2020). Thus, investment in the basic infrastructure of shelters with supportive services is still needed. In addition, we recognize the interdependence between the “culture of care” at Lotus House and the variety and depth of the services provided. Enhanced services at shelters across the nation, alongside the creation and maintenance of supportive cultures, is of critical importance (Burt et al., 2010).

Finally, the results from this study are being used by Lotus House staff to address factors participants cited as concerns, while continuing to deliver identified service strengths. According to Lotus House leadership, this study has been used to shape the current and future operations, policies, and procedures of Lotus House. For example, the present paper has been circulated among Lotus House leadership and staff, resulting in conversations and efforts to continuously improve the culture and services offered, with the objective of developing a national model. Inspired by this study, Lotus House is currently conducting a national survey of women's shelters to deepen their connections, gather and share information on challenges and best practices, and advance the status of women and children experiencing homelessness. These efforts align with CBPR goals of developing research that contributes to the mobilization of a collective action strategy, leading to system changes (Baum et al., 2006; Newell & South, 2009; Vaughn et al., 2018).

### 4.3 Limitations

There are a series of limitations to address. Although it is advantageous to use focus groups to explore shared and unshared perceptions and experiences among group members, a disadvantage to group discussion is the possibility that one or more participants will dominate the conversation, making their views more prevalent within the analyses (Litosseliti, 2003). To this end, a particular perspective or opinion could have been misconstrued as shared by the entire group, which could happen when members who disagree stay quiet (Litosseliti, 2003). In the two situations where individuals began to dominant the discussion, the experienced facilitators used discussion redirection and using non‐verbal techniques to shift the focus (turning body slightly; Sim & Waterfield, 2019). Another limitation inherent to qualitative methods is that the sample is not representative of all women experiencing homelessness. Therefore, findings may not be generalizable to different samples of women who experience homelessness (Maxwell & Chmiel, 2014). Notably, this study was specifically focused on the experiences of sheltered women without children. The sample also represented only program completers who transitioned into stable housing after utilizing a unique shelter service in a specific geographic location.

Next, questions may arise about how trustworthy or valid the present research is. Because the research was conducted at Lotus House, rather than an independent facility, participants may have been hesitant to describe Lotus House in negative ways. There are two ways we protected against this concern. First, the instructions for the focus groups explicitly stated that we were interested in both the pros and cons of Lotus House, and that there were no right or wrong answers to any of the questions; we simply needed honest answers to improve the Lotus House experience for future guests. Second, Lotus House leadership were not present for the focus groups, reducing demand characteristics. Because we did hear comments critical of Lotus House during the focus groups, we can infer that at least some participants did not experience pressure to respond in socially desirable ways.

A final limitation of the present study is that we only examined the perceptions of former guests, and not shelter staff. In typical research examining organizational culture, culture is measured using organizational members at all levels (Hofstede et al., 1990). While our focus groups included former guests who had become staff at Lotus House (including those who were presently on staff), we did not ask them questions about their perceptions of Lotus House as staff members. This omission results from the fact that we did not directly set out to study Lotus House culture, but the general experiences and perceptions of guests; features of organizational culture spontaneously emerged from the focus groups. Future work on shelter culture should include shelter staff and leadership as well as guests.

### 4.4 Future directions

The ongoing study of the organizational culture of shelters is critical for the development of inclusive, effective, safe, and supportive shelter environments. Future directions should include the development of a scale that measures “culture of care” and integration of the idea of a “culture of care” into the organizational literature. In addition, future studies should examine the antecedents (e.g., leadership) and consequences (e.g., successful re‐housing and psychological symptoms) of a culture of care through quantitative and longitudinal methods, comparing across shelters. The discovery of a “culture of care” in shelters should also inspire the examination and implementation of a “culture of care” in other nonprofit settings and other settings in which the individuation, dignity, healing, and belonging of organizational members is of primary importance. In future implementations of a culture of care, the causal effects of culture on the well‐being of organizational members should be examined experimentally.

Finally, while there were questions in the interview protocol asking about the gender‐specific needs and experiences of the former guests, that data was too extensive to include in this paper. We did, however, find that women's experiences as guests at Lotus House took gender‐specific forms (e.g., the feeling of sisterhood). Therefore, future research should directly examine the extent to which a culture of care emerges in and is useful to members of women‐only compared to men‐only and mixed‐sex shelters and other organizations.

### PEER REVIEW

The peer review history for this article is available at https://publons.com/publon/10.1002/jcop.22579.

## Footnotes

1 Lotus House refers to all sheltered women, youth and children during their stay as “guests,” emphasizing the welcoming and voluntary nature of their participation.

2 While Lotus House is free, it is not without responsibility. To ensure they are able to shelter as many women as possible, sheltered women are asked not to spend more than $100 per month from any income and funds received so that they will have the requisite nest egg needed to make their move from the shelter as soon as reasonably possible.

## APPENDIX A

**Focus Group questioning route**

Thank you for agreeing to be in our study. I will be asking you a series of questions about your beliefs, your experience being homeless, and about being a guest in a shelter. Please be as honest as possible—our answers are confidential. There are no right or wrong answers—just your opinions. And, you don't have to share any thoughts or experiences unless you are comfortable doing so. Remember, the information you give will not include your name and is not tied back in any way to the survey you just completed.

1. Tell me about **your experience** being at the Lotus House.
  *Prompt:*
  a. Let's start with what was best about it?
  b. Best experience here?
2. What **services** were most helpful to you?
3. Would it have been better for you if you could have gone straight to your own apartment?
  *Prompt:*
  a. Benefits of LH experience
  b. Disadvantages of LH experience
4. Tell me about **safety**. Did you feel safe while at the Lotus House?
  *Prompt:*
  a. Where did you feel safe/not safe?
  b. What happened that made you feel/not feel safe?
5. Where did you sleep while experiencing homelessness?
6. Did you feel **safe** while you were homeless, before coming in to Lotus House?
  *Prompt:*
  a. Where did you feel safe/not safe?
  b. What happened that made you feel/not feel safe?
7. Before coming in, while homeless did you feel that you needed to **protect** yourself in some way?
  *Prompt:*
  a. How did you protect yourself?
  b. Was it possible to stay safe?
  c. How? When?
8. As a woman did you have any specific challenges or needs while experiencing homelessness?
  a. What specific challenges did you face?
  b. What specific needs did you have?
9. Besides Lotus House, how many of you spent time at **other** shelters? Did you feel protected from harm while you were there?

*Prompt:*

a. More/less than on the street?
b. All shelters the same?
c. Which ones better/worse?
d. Why? (vary by shelter)?

10. Now let's talk about the **ideal** shelter. Describe to me what it should be like for women.
  a. What would you have wanted?
  b. How would you want to be treated?
11. We started by talking about the Lotus House so let's wrap up with a bit more about the Lotus House. How might the Lotus House be a **better shelter** for its guests? What needs to be added or improved?
12. Is there **anything else** anyone wants to add? What did I not ask you that you would like to share?
